# ISWI Regulates Higher-Order Chromatin Structure and Histone H1 Assembly In Vivo

**DOI:** 10.1371/journal.pbio.0050232

**Published:** 2007-08-28

**Authors:** Davide F. V Corona, Giorgia Siriaco, Jennifer A Armstrong, Natalia Snarskaya, Stephanie A McClymont, Matthew P Scott, John W Tamkun

**Affiliations:** 1 Department of Molecular, Cell, and Developmental Biology, University of California Santa Cruz, Santa Cruz, California, United States of America; 2 Department of Developmental Biology, Stanford University School of Medicine, Stanford, California, United States of America; 3 Department of Genetics, Stanford University School of Medicine, Stanford, California, United States of America; 4 Department of Bioengineering, Stanford University School of Medicine, Stanford, California, United States of America; 5 Howard Hughes Medical Institute, Stanford University School of Medicine, Stanford, California, United States of America; University of California San Diego, United States of America

## Abstract

Imitation SWI (ISWI) and other ATP-dependent chromatin-remodeling factors play key roles in transcription and other processes by altering the structure and positioning of nucleosomes. Recent studies have also implicated ISWI in the regulation of higher-order chromatin structure, but its role in this process remains poorly understood. To clarify the role of ISWI in vivo, we examined defects in chromosome structure and gene expression resulting from the loss of *Iswi* function in *Drosophila*. Consistent with a broad role in transcriptional regulation, the expression of a large number of genes is altered in *Iswi* mutant larvae. The expression of a dominant-negative form of ISWI leads to dramatic alterations in higher-order chromatin structure, including the apparent decondensation of both mitotic and polytene chromosomes. The loss of ISWI function does not cause obvious defects in nucleosome assembly, but results in a significant reduction in the level of histone H1 associated with chromatin in vivo. These findings suggest that ISWI plays a global role in chromatin compaction in vivo by promoting the association of the linker histone H1 with chromatin.

## Author Summary

Chromatin-remodeling factors such as ISWI play a role in transcription and other nuclear processes by altering the structure and positioning of nucleosomes (the protein–DNA complexes that organize chromatin). Recent studies have suggested that chromatin-remodeling factors can also influence higher-order chromatin structure, but how they do this is not well understood. Using Drosophila melanogaster as a model organism, we investigated the role of ISWI in gene expression and the regulation of chromosome structure in higher eukaryotes. Loss of ISWI alters the expression of a large number of genes. The loss of ISWI function also causes dramatic alterations in higher-order chromatin structure—including the decondensation of mitotic and polytene chromosomes—accompanied by a striking reduction in the amount of the linker histone H1 associated with chromatin. Based on these findings, we propose that ISWI plays a global role in chromosome compaction by promoting the association of a linker histone with chromatin.

## Abstract 3

ISWI is a chromatin-remodeling factor that recruits linker histone H1 into chromatin, thus altering the structure and positioning of nucleosomes and the degree of chromatin compaction.

## Introduction

The packaging of DNA into chromatin is critical for the organization and expression of eukaryotic genomes. The basic unit of chromatin structure—the nucleosome—can repress transcription by blocking the access of transcription factors and other regulatory proteins to DNA [1]. Interactions between nucleosomes lead to the formation of 30-nm fibers, which can be further packaged into increasingly compact structures [2–4]. The regulation of higher-order chromatin structure is critical for chromosome condensation and segregation during mitosis and meiosis [5,6]. A growing body of evidence suggests that chromatin folding or looping is also important for the regulation of enhancer–promoter interactions and the subdivision of chromosomes into discrete functional domains [7]. The molecular mechanisms used to regulate chromatin structure have therefore been the topic of extensive study.

The repressive effect of chromatin on transcription is regulated via two general mechanisms: ATP-dependent chromatin remodeling and the covalent modification of histones. Chromatin remodeling reactions—including changes in the structure and spacing of nucleosomes—are catalyzed by ATPases that often function as subunits of large complexes, including the SWI/SNF, Imitation SWI (ISWI), and CHD complexes [8]. Histone-modifying enzymes alter the acetylation, methylation, phosphorylation, or ubiquitinylation of N-terminal histone tails and other regions on the surface of the nucleosome; these modifications modulate interactions between nucleosomes and a wide variety of structural and regulatory proteins [9]. Both histone-modifying enzymes and chromatin-remodeling complexes can be targeted to specific promoters by gene-specific or general transcription factors. By locally altering the structure or positioning of nucleosomes, histone-modifying enzymes and chromatin-remodeling complexes can activate or repress transcription of specific genes [1]. The coordinated activities of histone-modifying and chromatin-remodeling enzymes are therefore critical for transcription in a chromatin environment.

Interactions between histone-modifying and chromatin-remodeling enzymes can have profound effects on higher-order chromatin structure [10], as illustrated by recent studies of the *Drosophila* chromatin-remodeling factor ISWI. ISWI functions as the ATPase subunit of at least three distinct chromatin-remodeling complexes: ACF, CHRAC, and NURF [11,12]. These complexes use the energy of ATP hydrolysis to slide nucleosomes and alter the spacing of nucleosome arrays [12]. The loss of *Iswi* function in the larval salivary gland leads to the dramatic decondensation of a specific chromosome: the male X [13]. Similar defects in the structure of the male X chromosome are caused by loss-of-function mutations in *E(bx),* the gene encoding the largest subunit of NURF (NURF301) [14]. These findings suggest that ISWI plays a relatively global role in chromatin compaction in vivo.

The unusual sensitivity of the male X chromosome to the loss of ISWI function suggests that changes in chromatin structure that accompany dosage compensation might regulate the ability of ISWI to remodel chromatin in vivo. In *Drosophila,* dosage compensation is dependent on an RNA–protein complex that contains the MOF histone acetyltransferase [15,16]. This complex is specifically targeted to the male X chromosome, leading to the widespread acetylation of lysine 16 of the histone H4 tail (H4K16) by MOF [17]. This covalent modification is restricted to the male X chromosome and is thought to “open” chromatin structure by disrupting interactions between adjacent nucleosomes, thereby leading to the increased transcription of X-linked genes in males [18,19]. Dosage compensation is necessary and sufficient for the chromosome defects observed in *Iswi* mutant larvae, and genetic interactions between ISWI and MOF are consistent with functional antagonism between the two proteins [20]. Furthermore, biochemical studies have shown that the acetylation of H4K16 inhibits interactions between ISWI and its nucleosomal substrate in vitro [19–22]. Based on these observations, it has been proposed that the acetylation of H4K16 regulates chromatin compaction mediated by ISWI [19,20].

Although tremendous progress has been made toward understanding how ISWI alters the structure and positioning of nucleosomes, relatively little is known about how it alters higher-order chromatin structure and whether this activity is used to regulate transcription in a chromatin environment. To address these issues, we characterized defects in chromatin structure and gene expression resulting from the loss of *Iswi* function in *Drosophila*. Here we report that ISWI plays a surprisingly global role in the regulation of higher-order chromatin structure and transcription in vivo. Loss of ISWI function leads to widespread changes in gene expression, including the derepression of numerous genes. Defects in chromosome structure resulting from the loss of ISWI function are accompanied by a dramatic reduction in the level of histone H1 associated with chromatin. These findings suggest that ISWI plays a global role in chromatin compaction in vivo by promoting the association of histone H1 with chromatin.

## Results

### ISWI Is a Global Regulator of Polytene Chromosome Structure

Although genetic studies have shown that ISWI regulates the structure of the male X chromosome, its role in this process has remained unclear. ISWI might specifically regulate the structure of the male X chromosome, perhaps by dampening the effect of H4K16 acetylation on higher-order chromatin structure. In support of this view, the acetylation of H4K16 by the dosage compensation machinery is both necessary and sufficient for the chromosome defects observed in *Iswi* mutant larvae [20]. Alternatively, ISWI might regulate the structure of all chromosomes, with the male X chromosome being exquisitely sensitive to the loss of ISWI function. This sensitivity could be due to negative regulation of ISWI function by H4K16 acetylation, an idea that is supported by in vitro studies demonstrating that H4K16 acetylation blocks interactions between ISWI and its nucleosomal substrate [19,20]. To distinguish between the two models, we further investigated chromosome defects resulting from the loss of ISWI function in vivo.

**Figure 1:**
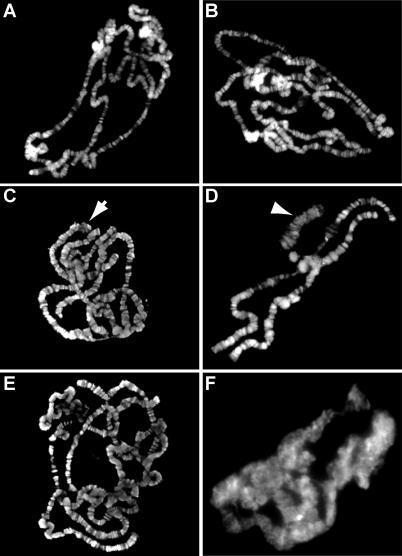
ISWI Plays a Global Role in Chromosome Compaction In Vivo Polytene chromosomes were prepared from larval salivary glands and stained with DAPI. Wild-type female (A) and male (B) polytene chromosomes exhibit normal morphology. The loss of zygotic *Iswi* function in *Iswi^1^*/*Iswi^2^* larvae causes the decondensation of the X chromosome in male (D, arrowhead) but not female (C, arrow) larvae. Expression of wild-type ISWI does not dramatically alter chromosome morphology (E). By contrast, the expression of a dominant-negative ISWI protein (ISWI^K159R^) in salivary gland nuclei causes the dramatic decondensation of all chromosomes (F).

Like many *Drosophila* genes, *Iswi* is expressed at high levels both maternally and zygotically; we therefore suspected that the high maternal contribution of *Iswi* gene products to the unfertilized egg might mask phenotypes resulting from the loss of zygotic *Iswi* function [13]. Our previous genetic studies of ISWI relied on two null alleles, *Iswi^1^* and *Iswi^2^* [13,20]. Individuals that are homozygous or trans-heterozygous for these alleles survive until late larval development, presumably due to the high maternal contribution of ISWI [13]. Consistent with this possibility, we detected low levels of ISWI in the salivary glands of *Iswi^1^*/*Iswi^2^* larvae by protein blotting (data not shown). Thus, the striking defects in the structure of the male X chromosome observed in these individuals [13] (Figure 1) did not reflect the true null phenotype of *Iswi* mutations. We therefore examined the consequences of further reducing *Iswi* function by producing a dominant-negative form of the ISWI protein, ISWI^K159R^.

The *Iswi^K159R^* mutation eliminates the ATPase activity of ISWI without affecting its ability to interact with other proteins; as a result, the expression of ISWI^K159R^ has very strong dominant-negative effects on ISWI function in vivo [13]. The expression of a GAL4-responsive *Iswi^K159R^* transgene (*UAS-Iswi^K159R^*) in salivary gland nuclei under the control of an *ey-GAL4* driver led to the dramatic decondensation of the X chromosome and the autosomes in both sexes (Figure 1F and data not shown). By contrast, the production of a variety of other proteins, including ISWI, GFP, and a dominant-negative form of another chromatin-remodeling factor (BRM^K804R^) did not cause similar defects in chromosome structure in either sex (Figure 1E and data not shown). These findings suggested that ISWI plays a global role in the regulation of polytene chromosome structure, as opposed to acting specifically on the male X chromosome.

### ISWI Is Required for the Generation or Maintenance of Higher-Order Chromatin Structure in Diploid Cells

**Figure 2:**
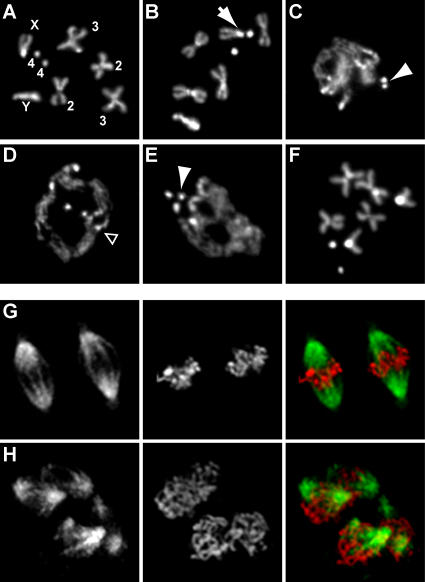
ISWI Regulates Chromosome Structure in Diploid Cells (A–F) Metaphase chromosome spreads isolated from neuroblasts of third-instar larvae grown at 18 °C. (A) Wild-type male metaphase chromosomes. (B) Metaphase chromosomes isolated from male *Iswi^1^*/*Iswi^2^* mutant larvae show normal condensation of the X chromosome (arrow). By contrast, mitotic chromosomes (C–E) of larval neuroblasts producing dominant-negative ISWI (ISWI^K159R^) appear much less condensed than normal. In these chromosomes, euchromatin is highly decondensed, whereas heterochromatic regions, such as centromeres (open arrowhead in [D]) and the fourth chromosomes (arrowheads in [C] and [E]), appear to condense normally. The production of dominant-negative BRM (BRM^K804R^) does not alter the morphology of mitotic chromosomes (F). (G and H) Nuclear cycle 12 embryos grown at 25 °C were stained for α-tubulin (green) and DAPI (red). Metaphase chromosomes in control +; *da-GAL4*/*T(2;3)B3 CyO, TM6B, Tb* embryos (G) appear normally condensed, while *Iswi^2^/+*; *UAS*-*Iswi^K159R^*/*da-GAL4* embryos (H) show abnormal chromosome morphology and spindle formation.

Although salivary gland polytene chromosomes provide a useful model for studying interphase chromosomes, their structure is unusual in many respects. Unlike chromosomes in diploid cells, the structure of polytene chromosomes is dependent on physical interactions between hundreds of sister chromatids formed by DNA replication in the absence of cell division [23]. This led us to wonder whether ISWI affects an aspect of chromatin organization unique to polytene chromosomes, as opposed to a more general aspect of higher-order chromatin structure. To investigate this issue, we examined whether the loss of ISWI function alters the structure of chromosomes in larval neuroblasts, a diploid cell type that is particularly well suited for cytological studies [24]. To allow observation of fully condensed chromosomes, neuroblasts were arrested in metaphase with colchicine. Mitotic chromosomes prepared from neuroblasts of wild-type and *Iswi^1^*/*Iswi^2^* third-instar larvae were indistinguishable (Figure 2A and 2B). Again, we suspected that the lack of a discernable phenotype might be due to the high maternal contribution of ISWI, since low levels of ISWI can be detected in extracts of neuroblasts by protein blotting (data not shown). We therefore expressed ISWI^K159R^ in neuroblasts of third-instar larvae using a *da-GAL4* transgene.

The expression of ISWI^K159R^ in larval neuroblasts dramatically altered the structure of metaphase chromosomes (Figure 2C–2E). Chromatin appeared highly decondensed and disorganized, and sister chromatids were often indistinguishable from each other. Loss of *Iswi* function most strongly affected euchromatic regions, which appeared hazy and diffuse. The effect on heterochromatic regions was much less striking, as evidenced by the relatively normal condensation of the largely heterochromatic fourth and Y chromosomes (Figure 2C–2E). Similar phenotypes were not observed in larvae expressing a variety of control proteins, including GFP, ISWI, and BRM^K804R^ (Figure 2F and unpublished data). The chromosome defects resulting from the expression of ISWI^K159R^ were highly penetrant. Thus, ISWI is required for the generation or maintenance of higher-order chromatin structure in both polytene and diploid cells.

Based on the known biochemical activities of ISWI, it is tempting to speculate that it regulates higher-order chromatin structure by altering the spacing or fluidity of nucleosome arrays. However, the chromosome defects observed in *Iswi* mutants might be a secondary consequence of changes in gene expression resulting from the loss of ISWI function. This is not a trivial concern, since salivary gland and neuroblast chromosomes cannot be examined until relatively late stages of larval development, approximately 5 d after fertilization. To help exclude this possibility, we examined whether the expression of ISWI^K159R^ alters chromosome structure in the early embryo. To minimize the maternal contribution of ISWI, we expressed ISWI^K159R^ in embryos produced by heterozygous *Iswi^2^* females at 25 °C. The GAL4 system used to drive the expression of ISWI^K159R^ is inherently temperature sensitive; much stronger phenotypes are observed at 25 °C than at 18 °C [25]. At 18 °C, individuals expressing ISWI^K159R^ under the control of a *da-GAL4* driver survive until late larval stages, but at 25 °C they fail to complete embryogenesis (data not shown). Defects in chromosome structure resulting from the loss of *Iswi* function were examined by staining fixed embryos with DAPI and antibodies against α-tubulin. Defects in chromosome condensation and the organization of the mitotic spindle were observed as early as nuclear cycle 12, shortly after the onset of zygotic transcription (Figure 2). Similar defects were not observed in control embryos expressing a variety of control proteins, including BRM^K804R^ (data not shown). These findings suggest that ISWI directly regulates higher-order chromatin structure.

### ISWI Plays Relatively Global Roles in Transcriptional Activation and Repression In Vivo

**Figure 3:**
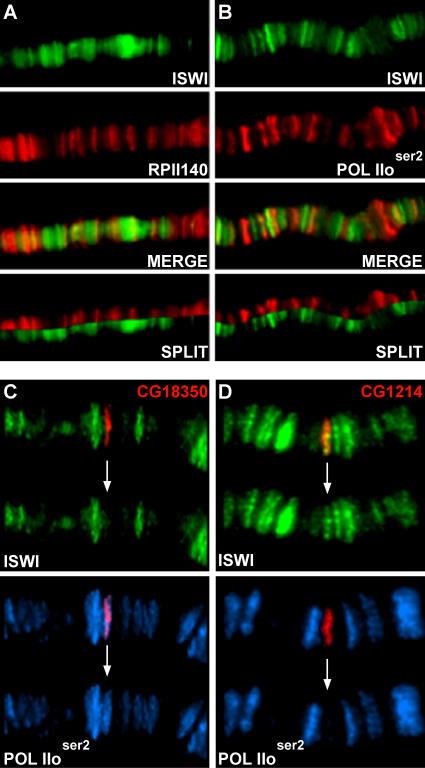
ISWI Plays a Relatively Global Role in Transcriptional Activation and Repression In Vivo (A and B) ISWI is associated with hundreds of euchromatic sites in a pattern that is largely complementary to that of Pol II. (A) ISWI (green) and Pol II (red) were detected using antibodies against ISWI and the second largest subunit of Pol II (RPII140). The distributions of ISWI and RPII140 are largely nonoverlapping, as observed in the split image. (B) ISWI (green) and elongating Pol II (red) were detected using antibodies against ISWI and the antibodies against Pol II hyperphosphorylated on Ser2 of the CTD (Pol IIo^ser2^). Very little overlap is observed between ISWI and elongating Pol II, as observed in the split image. (C and D) Analysis of physical interactions between ISWI and its potential target genes in polytene chromosomes by immuno-FISH. Wild-type polytene chromosomes were stained with antibodies against ISWI (green) and the elongating form of Pol II (Pol IIo^ser2^) (blue). FISH analysis identifies the locus of genes CG18350/*Sxl* (C) and CG1214/*ru* (D) in red. CG18350, which is expressed at reduced levels in *Iswi* mutants, localizes with Pol IIo^ser2^ but does not co-localize with ISWI, as indicated by the arrows. CG1214, which is expressed at elevated levels in *Iswi* mutants, co-localizes with ISWI but not with Pol IIo^ser2^, as indicated by the arrows.

ISWI is associated with hundreds of euchromatic sites of *Drosophila* polytene chromosomes in a pattern that is largely complementary to that of RNA polymerase II (Pol II) [13] (Figure 3A). This bias is even more pronounced when the distribution of ISWI is compared to that of the elongating form of Pol II (Pol IIo^ser2^) (Figure 3B). To determine if ISWI plays a global role in transcriptional repression, we monitored changes in gene expression resulting from the loss of *Iswi* function in male and female third-instar larvae using whole-genome microarrays. Of nearly 15,000 genes analyzed, the expression of approximately 500 changed at least 2-fold in mutant male or female larvae relative to wild-type (Tables 1 and S1). Consistent with a predominant role for ISWI in transcriptional repression, nearly 75% of the genes are expressed at higher levels in *Iswi* mutants. The genes affected in *Iswi* mutants appear to be randomly distributed between the X chromosome and autosomes in both sexes and represent a functionally diverse group of genes with no obvious common properties or involvement in the regulation of higher-order chromatin structure (Tables 1 and S1).

**Table 1:**
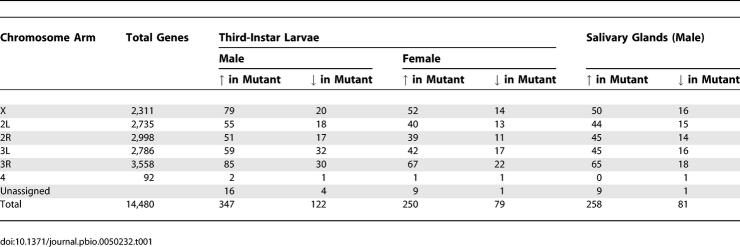
Number of Genes That Exhibit 2-Fold or Greater Changes in Expression in *Iswi^1^*/*Iswi^2^* Larvae

To address whether ISWI directly regulates the genes identified in our microarray studies, we examined whether it is physically associated with 16 of its potential target genes in salivary gland chromosomes. After staining polytene chromosomes from wild-type larvae with antibodies against ISWI and the elongating form of Pol II (Pol IIo^ser2^), the positions of potential target genes were identified by fluorescent in situ hybridization (FISH; Figure 3C and 3D). For these experiments, we selected genes that exhibited at least a 2.5-fold change in gene expression in *Iswi* mutant versus wild-type male larvae. ISWI was not associated with five of seven genes that are expressed at reduced levels in the salivary glands of *Iswi* mutant larvae (Figure 3C and unpublished data), suggesting that ISWI may indirectly activate their expression. By contrast, for eight of nine potential targets of repression examined, ISWI co-localized with the FISH signal, suggesting that it may directly repress the transcription of these genes (Figure 3D and unpublished data). These findings—together with the results of our microarray experiments and the preferential association of ISWI with weakly transcribed or silent regions of chromatin—is consistent with a predominant role for ISWI in transcriptional repression.

**Figure 4:**
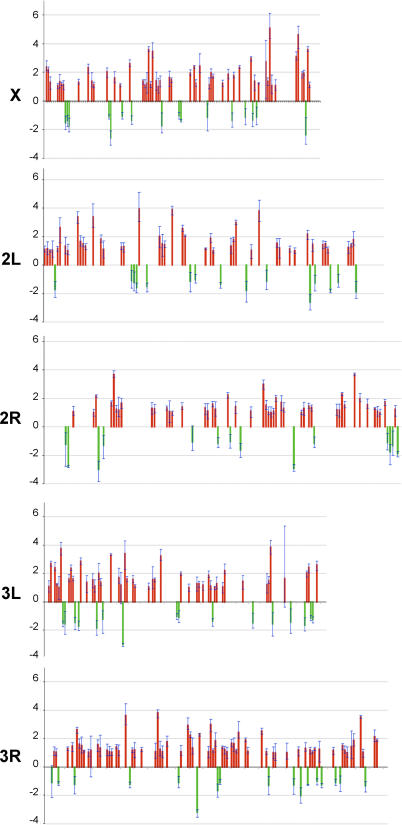
Genes That Exhibit Altered Expression in ISWI Mutants Are Broadly Distributed throughout the Genome The positions of euchromatic genes that exhibit a 2-fold or greater change in expression in the salivary glands of *Iswi* mutant males are shown. The *x-*axis corresponds to the relative cytological positions of genes on the X chromosome (X) and the left or right arms of the second (2L and 2R) and third (3L and 3R) chromosomes. Red and green bars mark genes that are repressed and activated by ISWI, respectively. The fold change in gene expression (log_2_) in *Iswi* mutants relative to wild-type is shown on the *y-*axis. The chromosomes are drawn to scale based on the amount of euchromatin found on each arm.

The changes in gene expression observed in *Iswi* mutants could be a consequence of global changes in chromatin compaction that increase the access of transcription factors or RNA polymerase to the DNA template. If this hypothesis is correct, one would expect that the loss of *Iswi* function in the salivary gland of male larvae would have a greater effect on the expression of X-linked genes than autosomal genes. We therefore examined the effect of *Iswi* mutations on gene expression in the salivary glands of third-instar larvae. In the salivary glands of *Iswi^1^/Iswi^2^* males, the expression of 339 genes changed at least 2-fold relative to wild-type; 76% of these genes were expressed at higher levels because of the loss of ISWI function (Tables 1 and S2). The potential targets of ISWI regulation in the salivary gland appear to be randomly distributed between the X chromosome and autosomes (Tables 1 and S2; Figure 4). Furthermore, the magnitude of the changes in the expression of potential ISWI target genes does not vary significantly as a function of their chromosomal locations (Figure 4; Table S2). Thus, the dramatic alterations in the structure of the male X chromosome in *Iswi* mutants are not accompanied by similarly dramatic changes in the expression of X-linked genes.

### ISWI Regulates the Structure of Autosomes via a Mechanism That Is Independent of H4K16 Acetylation

**Figure 5:**
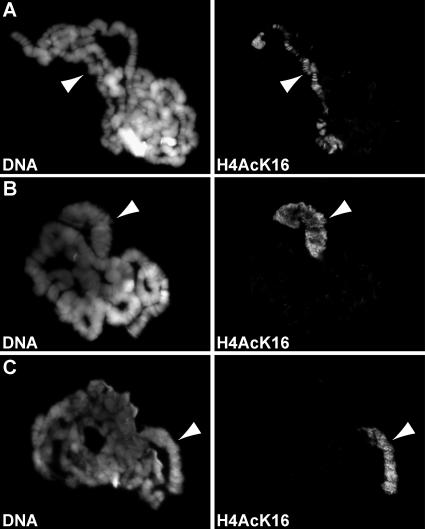
H4K16 Acetylation Is Not Affected by Loss of ISWI Salivary gland polytene chromosomes from male third-instar larvae were stained with DAPI and an antibody that specifically recognizes acetylated H4K16 (H4AcK16). The male X chromosome is marked by arrowheads. The level of H4K16 acetylation on the X chromosome of wild-type (A) and *Iswi^1^/Iswi^2^* (B) larvae is similar. Chromosome decondensation resulting from the expression of ISWI^K159R^ in salivary gland nuclei is not due to the spread of H4K16 acetylation to the autosomes (C). All three images were captured using identical exposure times.

We next investigated the molecular basis of the chromosome defects resulting from the loss of ISWI function. ISWI and MOF (the histone acetyltransferase that acetylates H4K16) have opposite effects on chromatin structure, and genetic studies have revealed a strong functional antagonism between the two proteins [20]. Based on these observations, we suspected that ISWI might promote chromatin compaction by blocking the acetylation of H4K16. To investigate this possibility, we stained polytene chromosomes of wild-type and *Iswi* mutant larvae with antibodies that specifically recognize acetylated H4K16. The levels of H4K16 acetylation on the male X chromosomes of wild-type and *Iswi* mutant larvae appear similar (Figure 5A and 5B) [13]. Furthermore, the chromosome defects observed in larvae expressing ISWI^K159R^ are not due to the spread of H4K16 acetylation to the autosomes (Figure 5C). These findings suggest that ISWI can promote the formation of higher-order chromatin structure independently of H4K16 acetylation.

### ISWI Promotes the Association of the Linker Histone H1 with Chromatin

**Figure 6:**
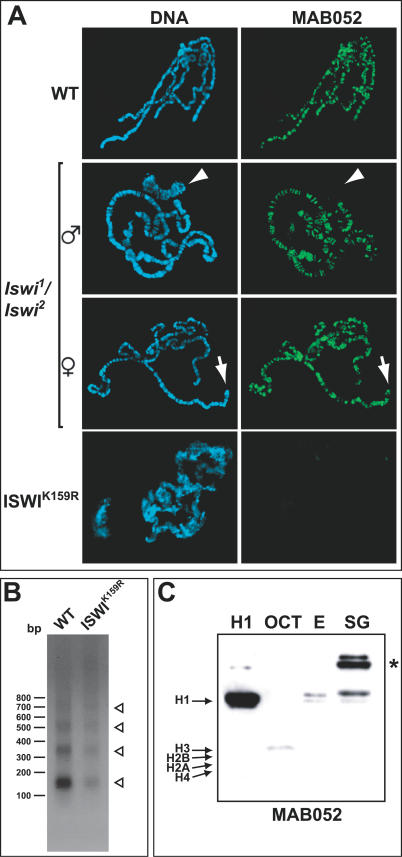
Loss of ISWI Results in Defects in Chromatin Assembly (A) The distribution of histones was analyzed on polytene chromosomes. Wild-type (WT) chromosomes immunostained with a pan-histone antibody (MAB052), which has been described as staining all histones, exhibit uniform histone staining of all arms. *Iswi^1^*/*Iswi^2^* male autosomes were uniformly stained, while staining of the X chromosome was dramatically reduced (arrowheads). *Iswi^1^*/*Iswi^2^* female chromosomes show uniform staining of all chromosomes, including the X chromosome, which is condensed normally (arrows). Chromosomes from cells that have produced a dominant-negative form of ISWI (ISWI^K159R^) have dramatically reduced staining. (B) Chromatin isolated from salivary glands of third-instar larvae was partially digested with microccocal nuclease. The open triangles indicate mono-, di-, tri-, and tetra-nucleosomal repeats. The approximate size of the DNA fragments can be visualized by referring to the 100-bp ladder marked alongside the gel. Comparison of digestion patterns of chromatin isolated from wild-type larvae and from larvae expressing ISWI^K159R^ reveals no obvious changes in nucleosome spacing or sensitivity to digestion. (C) Specificity of the reportedly pan-histone MAB052 antibody was analyzed by protein blotting: 20 ng of histone H1 (H1) purified from calf thymus, 1 μg recombinant *Drosophila* histone octamers (OCT), and embryo (E) and salivary gland (SG) extracts were resolved on a 12% SDS-polyacrylamide gel. The antibody, which is not really pan-histone, has a strong preference for histone H1 and only weakly recognizes recombinant *Drosophila* histone H3. In embryo and salivary gland extracts, the antibody exhibits a strong preference for histone H1, and also recognizes an uncharacterized band in salivary gland extracts (asterisk).

Biochemical studies have suggested several other mechanisms by which ISWI might regulate higher-order chromatin structure. For example, ISWI has been implicated in nucleosome assembly—a prerequisite for the formation of higher-order chromatin structure [26–28]. ISWI could also influence the packaging of nucleosome arrays by altering their spacing or fluidity. To determine if the loss of ISWI function causes gross defects in chromatin assembly, we stained wild-type and *Iswi* mutant larvae with a monoclonal antibody, MAB052, that has been reported to recognize the core histones H2A, H2B, H3, and H4 and the linker histone H1. As expected for a “pan-histone” antibody, MAB052 stained the X chromosome and autosomes of wild-type larvae in a pattern similar to that of DAPI-stained DNA (Figure 6A). In *Iswi^1^/Iswi^2^* larvae, MAB052 staining of the male X chromosome, but not the female X chromosome or autosomes of either sex, was dramatically reduced (Figure 6A). Furthermore, the chromosome defects resulting from the expression of ISWI^K159R^ were accompanied by loss of MAB052 staining from the autosomes as well as the X chromosome (Figure 6A). Similar results were obtained using a variety of fixation techniques, including treatment with either formaldehyde or citric acid (data not shown). The strong correlation between the loss of MAB052 staining and the severity of the chromosome defects observed in *Iswi* mutant larvae suggested that ISWI might indirectly regulate higher-order chromatin structure by promoting nucleosome assembly.

As an alternative approach for examining potential defects in nucleosome assembly resulting from the loss of ISWI function, we analyzed chromatin isolated from salivary glands of wild-type larvae and larvae expressing ISWI^K159R^ by partial digestion with micrococcal nuclease. As described above, the expression of ISWI^K159R^ in salivary gland nuclei leads to the decondensation of all chromosomes in both sexes. However, these defects are not accompanied by the appearance of subnucleosomal fragments characteristic of nucleosomes lacking histone H2A/B dimers or other core histones (Figure 6B). Furthermore, the loss of ISWI function did not lead to obvious changes in either the sensitivity of chromatin to micrococcal nuclease digestion or nucleosome spacing (Figure 6B and data not shown). Consistent with these observations, the decondensation of the male X chromosome in *Iswi^1^/Iswi^2^* larvae is not accompanied by decreased chromosomal levels of several core histones, including histone H4 acetylated on K16 (Figure 5), histone H3 trimethylated on K4 or K27 (Figure S1), and histone H2AvD (data not shown). These observations suggest that the chromosome defects observed in *Iswi* mutants do not result from gross defects in nucleosome assembly.

**Figure 7:**
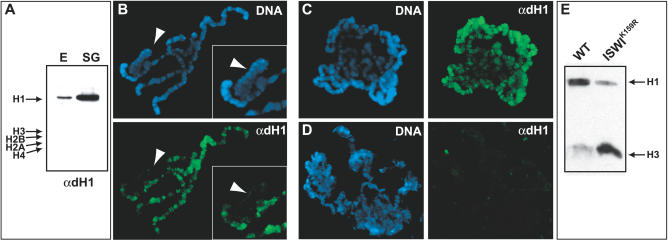
Loss of ISWI Results in Loss of Histone H1 (A) A polyclonal antibody (αdH1) specifically recognizes histone H1 in both embryo (E) and salivary gland (SG) extracts by protein blotting. (B) Immunostaining of male *Iswi^1^*/*Iswi^2^* polytene chromosomes with antibodies against histone H1 reveals that the levels of histone H1 are dramatically reduced on the decondensed X chromosome (arrowheads). The inserts show an enlarged image of the male X chromosome. (C and D) Wild-type chromosomes exhibit uniform staining with antibodies against histone H1 (C), whereas both the X chromosome and autosomes of larvae expressing ISWI^K159R^ have dramatically reduced staining (D). The images were captured with comparable exposure times. (E) Histone H1 levels are compared to histone H3 levels in salivary gland chromatin extracts by protein blotting using antibodies that specifically recognize *Drosophila* histone H1 [54] and histone H3 (Abcam, catalog number ab1791). Chromatin from larvae expressing ISWI^K159R^ was intentionally overloaded to ensure visualization of the histone H1 band. Consistent with the results presented in (D), the expression of ISWI^K159R^ leads to a significant (∼5-fold) reduction compared to wild-type (WT) in the ratio of histone H1 to histone H3 associated with chromatin.

To reconcile the seemingly contradictory results obtained using the above assays, we investigated the specificity of the supposedly pan-histone MAB052 antibody by protein blotting. Interestingly, we found that MAB052 has a strong preference for purified histone H1 (Figure 6C). The antibody only weakly detects histone H3 on a protein blot and does not recognize other recombinant core histones (Figure 6C). This strong preference for histone H1 was even more pronounced when extracts of *Drosophila* embryos and salivary glands were assayed by protein blotting using MAB052 (Figure 6C). We therefore repeated the above staining experiments using a polyclonal antibody specific for histone H1 (Figure 7). The loss of ISWI function does not significantly alter the total level of histone H1 in salivary glands as assayed by protein blotting (data not shown). However, as expected based on the specificity of MAB052, we found that histone H1 levels are dramatically reduced on the X chromosome of *Iswi^1^/Iswi^2^* males, but not the autosomes (Figure 7B). Furthermore, the expression of dominant-negative ISWI^K159R^ protein in salivary gland nuclei led to a dramatic reduction of histone H1 levels on all chromosomes of both sexes (Figure 7D and data not shown). In all cases, we observed an excellent correlation between the severity of the chromosome defects observed and the loss of histone H1 staining.

To confirm the above findings, we used a biochemical assay to determine whether the loss of ISWI function reduces the level of histone H1 associated with chromatin. Salivary glands were dissected from wild-type larvae and larvae expressing the ISWI^K159R^ protein and fixed with formaldehyde. Following chromatin extraction and the reversal of cross-linking, the levels of histone H3 and histone H1 associated with chromatin were assayed by protein blotting. As expected, the loss of ISWI function led to a significant and reproducible reduction in the ratio of histone H1 to histone H3 associated with chromatin (Figure 7E). These findings strongly suggest that defects in higher-order chromatin structure resulting from the loss of ISWI function are due to a failure to efficiently incorporate histone H1 into chromatin in vivo.

## Discussion

Most studies of ISWI complexes in *Drosophila* and other organisms have focused on their ability to alter the structure or spacing of nucleosomes, the fundamental unit of chromatin structure. Our findings reveal that ISWI also plays a global role in the regulation of higher-order chromatin structure. The *Iswi* mutations used in this study eliminate the function of multiple chromatin-remodeling complexes, including ACF, NURF, and CHRAC [11]. Which of these complexes are required for the formation of higher-order chromatin structure? Loss of function mutations in *Acf1—*which encodes a subunit protein shared by ACF and CHRAC—do not cause obvious defects in higher-order chromatin structure [27]. By contrast, loss of function mutations in *E(bx)—*which encodes a subunit specific to NURF—cause male X chromosome defects similar to those observed in *Iswi* mutants [14]. These findings suggest that ISWI modulates higher-order chromatin structure within the context of NURF, as opposed to ACF or CHRAC.

We observed a striking correlation between the severity of the chromosome defects resulting from the loss of ISWI function and the loss of the linker histone H1. This correlation suggests that ISWI regulates higher-order chromatin structure by promoting the association of histone H1 with chromatin. Histone H1 and other linker histones influence higher-order chromatin structure in vitro by stabilizing interactions between nucleosomes and chromatin fibers [29]. Although the ability of histone H1 to promote chromatin compaction in vitro is well established, its function in vivo has been a topic of considerable debate [30,31]. A protein with biochemical properties reminiscent of linker histones—HHO1—is present in budding yeast; surprisingly, HHO1 is not essential for viability in yeast, and *hho1* mutations have little effect on either gene expression or chromatin structure [32,33]. Genetic studies in *Tetrahymena* have suggested roles for linker histones in chromatin condensation and gene expression [34,35], but the relevance of these studies to histone H1 function in higher eukaryotes remains unclear. Studies of histone H1 function in higher eukaryotes have been complicated by the presence of redundant genes encoding histone H1 or histone H1 subtypes [36]. In spite of these difficulties, recent studies have revealed important roles for histone H1 in chromosome compaction in *Xenopus* and mice [37–40]. Thus, the chromosome defects observed in *Iswi* mutants could easily result from inefficient incorporation of histone H1 into chromatin.

How might ISWI promote the association of histone H1 with chromatin? Since ISWI is not required for histone H1 synthesis, ISWI may directly promote the assembly of chromatin containing histone H1 following DNA replication. Recent biochemical studies provide support for this possibility: ACF promotes the ATP-dependent assembly of H1-containing chromatin in vitro [26]. Loss of ACF1 function does not cause obvious changes in chromosome structure, however, suggesting that ACF either does not regulate higher-order chromatin structure in vivo or plays a redundant role in this process [27]. It remains possible that ISWI promotes the assembly of histone-H1-containing chromatin within the context of NURF or another chromatin-remodeling complex.

The ability to promote histone H1 assembly is not a common property of all chromatin-remodeling factors, as illustrated by recent biochemical studies of CHD1 [26]. Like ACF and other ISWI complexes, the CHD1 ATPase promotes the assembly of regularly spaced nucleosomes in vitro [26]. By contrast, CHD1 does not promote the incorporation of histone H1 during chromatin assembly in vitro [26]. These biochemical studies provide a plausible explanation for why the loss of ISWI function leads to the loss of histone H1 without causing dramatic changes in nucleosome assembly in vivo.

In other organisms, depletion of histone H1 leads to a significant decrease in the nucleosome repeat length [29], presumably because of the failure to efficiently incorporate histone H1 during replication-coupled chromatin assembly. By contrast, the loss of ISWI function in salivary gland nuclei leads to a decrease in the amount of histone H1 associated with chromatin without causing dramatic changes in nucleosome repeat length (Figure 6B). It is therefore tempting to speculate that ISWI promotes histone H1 incorporation via a replication-independent process. The association of histone H1 with chromatin is far less stable than that of core histones; histone H1 undergoes dynamic, global exchange throughout the cell cycle [41]. Photobleaching experiments in *Tetrahymena* and vertebrates have suggested that the majority of histone H1 molecules associated with chromatin are exchanged every few minutes [37,42–44], but little is known about the factors that regulate this process. Based on our findings, ISWI is an excellent candidate for a factor that regulates the dynamic exchange of histone H1 in vivo. Further work will be necessary to determine whether ISWI promotes histone H1 incorporation via replication-dependent or -independent mechanisms.

Our findings, together with previous studies, suggest that acetylation of H4K16 may regulate the association of linker histones with chromatin in vivo. The histone H4 tail is required for the nucleosome-stimulated ATPase activity of ISWI, and for its ability to slide nucleosomes and alter their spacing in vitro [21,22,45–47]. The region of the H4 tail that is critical for ISWI function in vitro is a DNA-bound basic patch (R_17_H_18_R_19_) adjacent to H4K16, the residue that is acetylated by the MOF histone acetyltransferase [21,22,47]. The acetylation of H4K16 interferes with the ability of ISWI to interact with the histone H4 tail and alter the spacing of nucleosome arrays in vitro [19,20,47]. Consistent with these findings, dosage compensation is necessary and sufficient for the decondensation of the X chromosome in *Iswi* mutant larvae, and genetic studies have revealed a strong functional antagonism between ISWI and MOF [20]. Thus, H4K16 acetylation may function as a switch that regulates the histone H1 assembly mediated by ISWI.

Our microarray studies revealed that ISWI is required for the proper expression of a large number of genes. These findings are consistent with numerous studies implicating ISWI in transcriptional regulation in vitro and in vivo [11,48,49]. Does ISWI modulate transcription by altering higher-order chromatin structure? We suspect that ISWI regulates transcription and higher-order chromatin structure via distinct mechanisms, since we observed no obvious correlation between the magnitude of the changes in gene expression and chromosome structure observed in *Iswi* mutant larvae. This is consistent with genetic studies in other organisms that have revealed that the loss of histone H1 does not cause dramatic changes in gene expression [33,34,37,40]. We also failed to observe a correlation between the magnitude of transcriptional derepression and gene size in *Iswi* mutant larvae (data not shown), as would be expected if ISWI relieved a general block to transcriptional elongation by Pol II. It should be noted, however, that relatively subtle, but biologically important, changes in gene expression may have escaped detection in our microarray studies. Further work will be necessary to clarify this issue and to determine whether ISWI regulates transcription and higher-order chromatin structure via distinct or related mechanisms.

## Materials and Methods

### *Drosophila* stocks and genetic crosses.

Flies were raised on cornmeal, agar, yeast, and molasses medium, supplemented with methyl paraben and propionic acid. Oregon R was used as the wild-type strain in all experiments unless otherwise noted. *Drosophila* strains were obtained from the Bloomington *Drosophila* Stock Center (http://flystocks.bio.indiana.edu/) and are described in FlyBase (http://www.flybase.org/), unless otherwise noted. The *Iswi* mutations used in this study are described elsewhere [13].

To generate larvae lacking zygotic *Iswi* function, *y w; Iswi^2^ sp; +/T(2;3) B3 CyO, TM6B, Tb* females were crossed to *Iswi^1^ Bc*/*SM5, Cy sp* males. The transheterozygous *Iswi* progeny resulting from this cross are referred to as *Iswi^1^/Iswi^2^* elsewhere in the text.

The GAL4 system [25] was used to drive the expression of ISWI^K159R^ and other proteins in the salivary gland and other tissues. Two GAL4 driver lines were used in this study: *da-GAL4* [50] and *ey-GAL4* [51]. *ey-GAL4* drives expression in the larval salivary glands and eye-antennal disc; *da-GAL4* is widely expressed at all stages of development. The UAS lines used in this study include (1) *Df(1)w67c2 y; P[w^+^, UAS-Iswi^K159R^-HA-6His] 11–4*/*TM6B, P[w^+^ Ubi-GFP], Tb* (referred to as *UAS-Iswi^K159R^*); (2) *w^1118^; P[w^+^ UAS-GFP.nls]* (referred to as *UAS-GFP*); (3) *w; P[w^+mC^ UAS-LacZ.B] 4-1-2* (referred to as *UAS-LacZ*); (4) *Df(1)w67c2 y; P[w^+^ UAS-brm^K804R^] 2–2* (referred to as *UAS-brm^K804R^*); (5) *Df(1)w67c2 y P[w^+^ UAS_G_-Iswi-HA-6His] 18* (referred to as *UAS-Iswi*); (6) *w; al b cn Iswi^2^ sp; P[w^+^, UAS-Iswi^K159R^-HA-6HIS] 11–4/T(2;3) B3 CyO, TM6B, Tb;* and (7) *w; P[w^+^, eyGAL4], P[w, UAS-Iswi^K159R^-HA-6His] 11–4*/*TM3, Sb*.

### Analysis of polytene chromosome structure.

Polytene chromosomes were isolated from salivary glands of third-instar larvae maintained at 18 °C. To examine the effect of *Iswi* mutations on polytene chromosome structure, salivary glands were isolated from *Iswi^1^/Iswi^2^* larvae. The effect of ISWI^K159R^ expression on polytene chromosome structure was examined by crossing *w; P[w^+^, UAS-Iswi^K159R^-HA-6His] 11–4*/*TM6B, P[w^+^Ubi-GFP], Tb* flies to *da-GAL4* or *w; P[w^+^, eyGAL4]* flies at 18 °C. Salivary glands were isolated from larvae bearing *UAS-Iswi^K159R^* in trans to *ey-GAL4* or *da-GAL4*. Salivary glands bearing *UAS-LacZ*, *UAS-GFP,* and *UAS-brm^K804R^* in trans to *da-GAL4* or *ey-GAL4* were analyzed for control experiments.

To analyze polytene chromosome structure, salivary glands were dissected in 0.7% NaCl and squashed in 1.85% formaldehyde/45% acetic acid. Slides were frozen in liquid nitrogen, air dried, and counterstained and mounted in Vectashield containing DAPI (Vector Laboratories, http://www.vectorlabs.com/).

Antibodies used in this study include affinity-purified rabbit anti-ISWI [52]; mouse anti-RPII140 [53]; rabbit anti-H4AcK16 (catalog number ab1762, Abcam, http://www.abcam.com/); rabbit anti-H3K27Me(3×) (catalog number 07–449, Upstate, http://www.upstate.com/); rabbit anti-H3K4Me(3×) (Upstate, catalog number 07–473); mouse anti-Pol IIo^ser2^ (Covance, http://www.covance.com/); mouse pan-histone monoclonal antibody MAB052 (catalog number MAB052, Chemicon, http://www.chemicon.com/); and rabbit anti-histone H1 [54]. Secondary antibodies were obtained from Jackson ImmunoResearch Laboratories (http://www.jacksonimmuno.com/). Slides were counterstained and mounted in Vectashield containing DAPI (Vector Laboratories). Immunostaining of wild-type polytene chromosomes with antibodies against ISWI and Pol II subunits was carried out as described previously [55]. For the staining of polytene chromosomes with monoclonal antibody MAB052 and antibodies against acetylated H4K16, trimethyl H3K27, trimethyl H3K4, and *Drosophila* histone H1, glands were dissected in 0.7% NaCl and fixed in 6 mM MgCl_2_/1% citric acid/1% Triton X-100 for 2 min. Chromosome preparations were analyzed using a Zeiss Axioskop 2 plus fluorescent microscope equipped with an Axioplan HRm CCD camera and Axiovision 4.2 software (Zeiss, http://www.zeiss.com/). To directly compare the chromosomal levels of acetylated H4K16 and histone H1 in wild-type and mutant larvae, chromosomes were squashed and processed in parallel, and images were captured using identical exposure times.

### Immuno-FISH.

The association of ISWI with nine potential targets of ISWI repression—CG7951 *(sima);* CG18166; CG7997; CG2162; CG1787 (*Hexo2*); CG7696; CG3765 *(Pde9);* CG1214 *(ru);* and CG12002 *(Pxn)*—and seven potential targets of ISWI activation—CG4690 *(Tsp5D);* CG18350 *(Sxl);* CG10934; CG8094 *(Hex-C);* CG1146; CG4281; and CG9531—was examined. cDNAs corresponding to each gene were obtained from the *Drosophila* Genomics Resource Center (https://dgrc.cgb.indiana.edu). Wild-type polytene chromosomes were stained with antibodies against ISWI and Pol IIo^ser2^ as described previously [55]. The chromosome squashes were then washed in PBS for 5 min, fixed in 3.7% formaldehyde/PBS for 10 min, and washed again in PBS for 5 min. FISH was subsequently carried out as described in Pimpinelli et al. [56], except that denaturation in 70% formamide/2× SSC was carried out for 8 min and probes were labeled using a biotin nick translation mix (Roche, http://www.roche.com/). Slides were counterstained and mounted in Vectashield containing DAPI (Vector Laboratories).

### Analysis of mitotic chromosomes from larval brains.

To examine the effect of loss of zygotic ISWI function on mitotic chromosome structure, neuroblasts were isolated from wild-type and *Iswi^1^/Iswi^2^* larvae. The effect of ISWI^K159R^ expression on chromosome structure was examined by crossing *w; P[w^+^, UAS-Iswi^K159R^-HA-6His] 11–4*/*TM6B, P[w^+^Ubi-GFP], Tb* flies to *da-GAL4* flies at 18 °C. Neuroblasts were isolated from larvae bearing *UAS-Iswi^K159R^* in trans to *da-GAL4*. Neuroblasts bearing *UAS-LacZ, UAS-GFP,* and *UAS-brm^K804R^* in trans to *da-GAL4* were analyzed for control experiments. Metaphase chromosomes were prepared from neuroblasts of third-instar larvae as described by Cenci et al. [57]. Slides were counterstained and mounted in Vectashield containing DAPI (Vector Laboratories). Chromosome preparations were analyzed using a Zeiss Axioskop 2 plus fluorescent microscope equipped with an Axioplan HRm CCD camera, and Axiovision 4.2 software (Zeiss).

### Analysis of chromosome defects in early *Drosophila* embryos.

To express *Iswi^K159R^* in heterozygous *Iswi^2^* embryos, *w; al b cn Iswi^2^ sp; P[w^+^, UAS-Iswi^K159R^-HA-6HIS] 11–4/T(2;3) B3 CyO, TM6B, Tb* females were crossed to *da-GAL4* males at 25 °C. Embryos were collected which were either +; *da-GAL4/T(2;3) B3 CyO, TM6B, Tb* or *al b cn Iswi^2^ sp/+; P[w^+^, UAS-Iswi^K159R^-HA-6HIS] 11–4/da-GAL4*. Embryos bearing *UAS-LacZ, UAS-GFP,* or *UAS-brm^K804R^* in trans to *da-GAL4* were analyzed for control experiments. Embryos were collected and fixed with methanol as described by Rothwell and Sullivan [58], stained with a mouse anti-α-tubulin antibody (catalog number T9026, Sigma-Aldrich, http://www.sigmaaldrich.com/), counterstained, and mounted in Vectashield containing propidium iodide (Vector Laboratories). Embryo preparations were analyzed using an inverted microscope (DM IRB, Leica Microsystems, http://www.leica-microsystems.com/) equipped with a laser confocal imaging system (TCS SP2, Leica Microsystems), and images were acquired and analyzed using Leica Microsystems confocal software version 2.61.

### Electrophoresis and protein blotting.

Proteins were extracted from *Drosophila* embryos as described in Srinivasan et al. [59]. To prepare salivary gland protein extracts, salivary glands were dissected from third-instar larvae in 0.7% NaCl; transferred to a microfuge tube containing 100 μl PBS, 0.8% NP-40, 1 mM DTT, and 1 mM PMSF; pelleted by centrifugation at 2,100*g* for 5 min at 4 °C; resuspended in boiling SDS–polyacrylamide gel electrophoresis (SDS-PAGE) loading buffer; homogenized with a pestle; and frozen in liquid nitrogen. Proteins were fractionated by SDS-PAGE and analyzed by protein blotting as described in Srinivasan et al. [59]. Primary antibodies were detected using horseradish-peroxidase-coupled secondary antibody (Bio-Rad, http://www.bio-rad.com/) and Super Signal chemiluminescent reagent (Pierce, http://www.piercenet.com/).

### Biochemical analysis of salivary gland chromatin.

To prepare salivary gland chromatin extracts, salivary glands were dissected from third-instar larvae in 0.7% NaCl; transferred to a microfuge containing 2% formaldehyde in 100 μl of 0.5× M buffer (10 mM Hepes-KOH [pH 7.6], 25 mM KCl, 5 mM MgCl_2_, 5% glycerol), 1 mM DTT, and 1 mM PMSF; and incubated for 15 min at room temperature. The cross-linking reaction was stopped with 125 mM glycine. Salivary glands were transferred to a microfuge tube containing 100 μl of M buffer, 0.8% NP-40, 1 mM DTT, and 1 mM PMSF; incubated on ice for 15 min; homogenized with a pestle; pelleted by centrifugation at 2,100*g* for 5 min at 4 °C; and resuspended in boiling SDS-PAGE loading buffer. Proteins were fractionated by SDS-PAGE and analyzed by protein blotting as described above using rabbit antibodies against rabbit *Drosophila* histone H1 [54] and histone H3 (Abcam, catalog number ab1791). Chemiluminescent signals were quantified using a Bio-Rad Molecular Imager.

### Analysis of chromatin by micrococcal nuclease digestion.

Partial micrococcal nuclease digestion of salivary gland chromatin was conducted using a modification of a protocol described in Cartwright et al. [60]. Chromatin was isolated from wild-type or *w; P[w^+^, ey-GAL4], P[w^+^, UAS-Iswi^K159R^-HA-6-His] 11–4*/*TM3, Sb* third-instar larvae. Salivary glands were dissected in M buffer (10 mM Hepes-KOH [pH 7.6], 25 mM KCl, 5 mM MgCl_2_, 5% glycerol) and transferred to 100 μl of M buffer, 0.5 mM PMSF, and 0.5 mM DTT. After addition of 4 μl of 20% NP-40, the samples were incubated on ice for 15 min and homogenized with a pestle. The samples were centrifuged at 2,100*g* for 5 min at 4 °C; washed with MNase buffer (M buffer, 2 mM CaCl_2_, 0.5 mM PMSF); centrifuged at 2,100*g* for 5 min at 4 °C; and resuspended in 200 μl of MNase buffer containing 10,180 units of micrococcal nuclease (USB, http://www.usbweb.com/). After digestion for 4 min at room temperature, the reaction was stopped by the addition of 200 μl of S buffer (20 mM Tris-HCl [pH 7.4], 200 mM NaCl, 2 mM EDTA, 2% SDS, 30 mM EGTA). Proteinase K was added to a final concentration of 1 mg/ml, and the samples were incubated for 1 h at 45 °C. Following phenol-chloroform extraction and the addition of 1 μl of glycogen (Roche), the DNA was ethanol precipitated and resuspended in 120 μl of RNase buffer (50 mM Tris-HCl [pH 7.5], 100 mM NaCl, 10 mM EDTA). RNase A (Sigma) was added to a final concentration of 250 μg/ml, and the samples were incubated for 1 h at 37 °C. Following another round of phenol-chloroform extraction and ethanol precipitation, DNA was resuspended in 10 μl of TE and analyzed by agarose gel electrophoresis.

### Microarray studies.

Microarrays containing ∼14,400 cDNA fragments representing over 14,000 different *Drosophila* genes were used to characterize changes in gene expression resulting from the loss of ISWI function. Control larvae were generated by crossing Oregon R males to *Df(1)w67c2 y* virgin females. *Iswi^1^*/*Iswi^2^* larvae were generated by crossing *w; al b cn Iswi^2^ sp; +/T(2;3) B3 CyO, TM6B, Tb* virgin females to *Iswi^1^ Bc*/*SM5, Cy sp* males. Male and female third-instar larvae of the appropriate sex and genotype were identified using the markers *y, Bc,* and *Tb*. RNA was isolated from third-instar larvae or dissected salivary glands by homogenization in Trizol reagent (Invitrogen, http://www.invitrogen.com/) followed by chloroform extraction and precipitation with isopropanol. Detailed protocols for the RNA isolation, cDNA synthesis, construction of microarrays, and hybridization are provided in Protocol S1.

Microarrays were scanned and analyzed using an Axon scanner and GenePix software (MDS Analytical Technologies, http://www.moleculardevices.com/). The data acquired with GenePix were uploaded to the Stanford MicroArray Database (http://genome-www5.stanford.edu/) and analyzed using Stanford MicroArray Database data analysis software. Data were normalized by ribosomal RNA inputs, by spot intensity/background readings, and by data taken from mitochondrial genes (i.e., *mt:ND3* and *mt:ND6*). Data obtained from triplicate hybridizations per experiment were filtered for measurement values with a regression correlation coefficient equal or greater than 0.6. Data that passed the spot criteria were filtered based on gene data values whose log_2_R/G normalized ratio (mean) was equal to or greater than four. Finally, data that passed all the above criteria were clustered using a non-centered Pearson correlation algorithm.

## Supporting Information

Figure S1 Chromosomal Levels of Histone H3 Are Not Affected by the Loss of ISWI Function Salivary gland polytene chromosomes from male *Iswi^1^/Iswi^2^* third-instar larvae were stained with DAPI and antibodies that specifically recognize trimethyl H3K27 (A) and trimethyl H3K4 (B). The male X chromosomes are marked by an arrowhead. The decondensation of the male X chromosome is not accompanied by obvious changes in the level of either histone modification.
(3.5 MB PDF)

Protocol S1 Additional Methods (45 KB DOC)

Table S1 Changes in Gene Expression Resulting from the Loss of ISWI Function in Whole *Drosophila* Larvae (1.8 MB XLS)

Table S2 Changes in Gene Expression Resulting from the Loss of ISWI Function in the Salivary Glands of *Drosophila* Larvae (110 KB XLS)

## Abbreviations

FISH: fluorescent in situ hybridization
H4K16: histone H4 lysine 16
ISWI: Imitation SWI
Pol II: RNA polymerase II
SDS-page: SDS–polyacrylamide gel electrophoresis
