# Supplementary material for: ISWI Regulates Higher-Order Chromatin Structure and Histone H1 Assembly In Vivo
Source: PLoS Biol. 2007 Aug 28;5(9):e232. doi: 10.1371/journal.pbio.0050232 (PMC1951781; doi:10.1371/journal.pbio.0050232)
Supplement: Supplementary file 1 [file pbio.0050232.sg001.pdf]

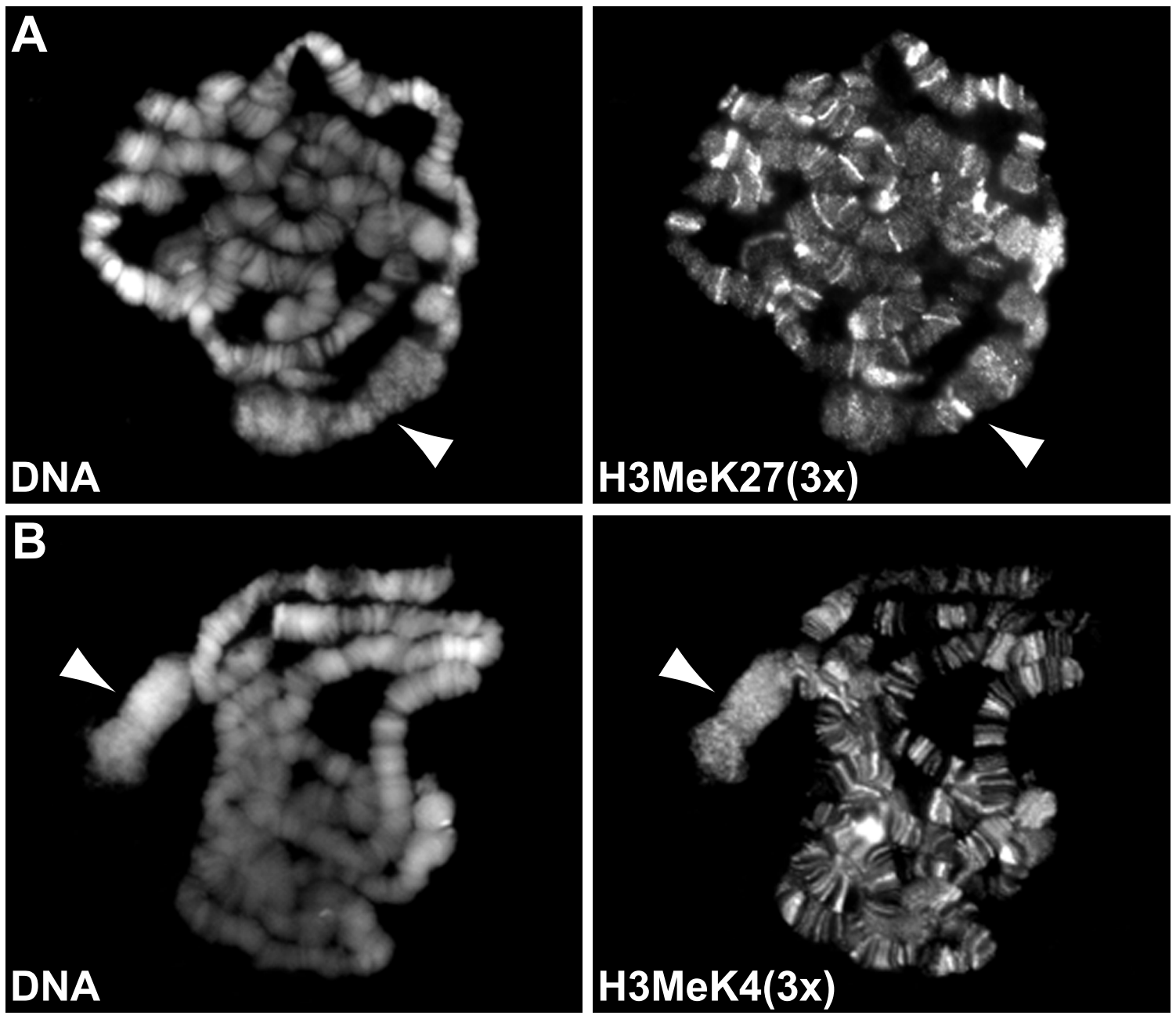
A
DNA
H3MeK27(3x)
B
DNA
H3MeK4(3x)
