# Supplementary material for: ISWI Regulates Higher-Order Chromatin Structure and Histone H1 Assembly In Vivo
Source: PLoS Biol. 2007 Aug 28;5(9):e232. doi: 10.1371/journal.pbio.0050232 (PMC1951781; doi:10.1371/journal.pbio.0050232)
Supplement: Supplementary file 2 [file pbio.0050232.sd001.doc]

## Construction of DNA microarrays

Microarrays were constructed with ~14,400 cDNA elements representing over 14,000 different *Drosophila* genes. The Incyte Easy-to-Spot Drosophila PCR Primer Set was used to obtain PCR-amplified DNA from genomic DNA. Using genomic sequence information from the Berkeley Drosophila Genome Project (BDGP; www.bdgp.org), Incyte selected ~14,000 primer pairs to amplify unique open reading frames from the *Drosophila* genome. Primer design consisted of two algorithm modules using public-domain genomic sequences (BDGP). Fragment selection was a comprehensive process designed to minimize homology between array elements. The Primer 3 Program (<http://www.genome.wi.mit.edu/genome_software/other/primer3.html>) was used for primer selection. The selection was designed to maximize the specificity of the PCR reaction. One exon from each gene was selected and primers were designed with the following parameters: 1) no intronic sequence allowed; 2) 58 – 68°C melting temperature; 3) 100-600bp amplicon sizes; 4) primers were blasted against the whole genome to determine potential false priming sites.

Standard PCR conditions were used to create PCR products on MJ Research thermocyclers. Each PCR product was purified by ethanol precipitation with 120mM sodium acetate, washed with 70% ethanol, air-dried and stored at –20°C. Each PCR product was checked by gel-electrophoresis. Approximately 14,400 PCR reactions (97%) yielded a single band of the predicted length. Purified PCR fragments were dissolved in 3xSSC buffer and spotted onto poly-L-lysine-coated glass slides (homemade) using an OmniGrid robot [GeneMachines]. The spotter utilizes 32 printing tips which simultaneously pick up ~1µl of solution from 386-well plates. After printing, the microarrays were rehydrated for 4 minutes in a humid chamber [Sigma] and then snap-dried for 3 seconds on a hot plate (75°C). The DNA was then UV-crosslinked to the surface by subjecting the slides to 65mJ of energy. The rest of the poly-L-lysine surface was blocked by a 20 minute incubation in a solution containing 6g of succinic anhydride dissolved in 335ml of 1-methyl-2-pyrrolidinone [Sigma] and 15ml of 1M sodium borate (pH8.0). Following the blocking reaction, the bound DNA was denatured by a 2 minute incubation in milli-Q H_2_O at 95°C. The slides were then transferred into a bath of 100% ethanol at room temperature for 2 minutes and then dried by centrifugation. Post-processed slides were stored at room temperature in a dessicator box.

## RNA isolation from salivary glands and whole larvae

To prepare salivary gland RNA, *ISWI^1^*/*ISWI^2^* larvae were generated by crossing *y w*; al b cn *ISWI^2^* *sp*; *+*/*T(2:3)CyO*,*TM6B*, *Tb* females to *ISWI^1^ Bc*/*SM5, Cy sp* males. Control larvae were generated by crossing Oregon R males to *Df(1)w67c2 y* virgin females. Male and female third instar larvae of the appropriate genotype were identified using the markers *y*, *Bc* and *Tb*. 25 salivary glands were dissected and pooled in an eppendorf with 60µl of 0.7% NaCl buffer to which 600µl of TRIzol reagent [Invitrogen] were added. The salivary glands were homogenized and centrifuged at 10,000 rpm for 1 minute. The small pellet was sheared by pipetting the supernatant and pellet through a 200 µl tip. Following the addition of 10µg of linear polyacrylamide [GenElute, Sigma] and 120 µl of chloroform, the sample was vortexed for 30 seconds and processed as described below.

RNA was prepared from whole larvae as follows. 25 male or female third instar larvae were homogenized in 1ml of TRIzol reagent in a 2ml glass dounce homogenizer by ten strokes of a B pestle followed by 10 strokes of an A pestle. The homogenate was centrifuged at 10K for 1 minute and supernatant was transferred to a new eppendorf tube. Following the addition of 20 µg of linear polyacrylamide and 200µl of chloroform the sample was vortexed for 30 seconds and processed as described below.

Both salivary gland and whole larval RNA preparations were then processed as follows. After centrifigution at 14,000 rpm for 5 minutes at room temperature, the supernatant was transferred to an eppendorf tube and 0.7 volume of isopropanol was added. After vortexing and overnight storage at -20°C, RNA was pelleted by centrifugation at 14,000 rpm for 30 minutes at 4°C. The pellet was washed with 75% cold ethanol, air dried, resuspended in RNAse-free water (15 µl for salivary gland and 50 µl for whole larval RNA preps) and incubated for 15 minutes at 37 °C. The quality and integrity of RNA samples were evaluated by formaldehyde agarose gel electrophoresis followed by staining with SYBR Gold [Molecular Probes].

*cDNA synthesis and microarray hybridization*

1µg of anchored oligo-dT primer [(dT)20-VN] [Operon, HPLC-purified] was annealed to 15µg of total RNA by heating the reaction to 70°C for 5 minutes in a total volume of 18.5µl followed by rapid cooling to room temperature. The reverse transcription reaction mix included 1µl of PowerScript reverse transcriptase [BD Biosciences], 1µl 30x dNTP mix (15 mM each of dATP, dCTP, dGTP; 100µM dTTP [Pharmacia] and 400µM aminoallyl-dUTP [Sigma], 6µl 5x reaction buffer, 3µl 0.1M DTT and 0.5µl RNasin [Promega]. Reactions were carried out at 42°C for 1.5 hours, after which the RNA was degraded by the addition of 3µl 2.5N NaOH followed by incubation at 70°C for 10 minutes. The samples were neutralized by the addition of 15µl of 2M HEPES and ethanol-precipitated with 0.3M NaOAc at –20°C overnight. After centrifugation and a 70% ethanol wash, samples were air-dried for 5 minutes and resuspended in 10µl of nuclease-free water [Ambion].

Cy3 and Cy5 dye aliquots [Amersham] were resuspended in 20µl of 0.1M NaHCO_3_ (pH9.0) and 10µl of dye were added to each sample. After incubating the samples in the dark for 1 hour at room temperature, unreactive NHS-ester Cy dyes were quenched by the addition of 10µl of 4M hydroxylamine [Sigma] followed by a 10 minute incubation in the dark to prevent cross coupling and samples were purified using the Qia-Quick PCR purification kit [QIAGEN]. An additional wash step with 35% guanidine HCl was performed to remove any unbound dye.

Purified, labeled probes were resuspended in TE buffer containing 3x SSC and 0.3% SDS. Cy3 and Cy5-labeled probes were mixed together; heated for 2 minutes at 100°C, cooled rapidly to room temperature, and applied to the arrays. The arrays was placed in a sealed, humidified chamber [Incyte] and hybridized for 16 hours in a 65°C oven. After hybridization, the arrays were washed for 2 minutes in 2x SSC/ 0.03% SDS, 1x SSC and twice in 0.2x SSC, after which they were dried by centrifugation.
